# Micronutrient Biosynthesis Potential of Spontaneous Grain Fermentation Microbiomes

**DOI:** 10.3390/ijerph192416621

**Published:** 2022-12-10

**Authors:** Margaret I. Dania, Bahram Faraji, James Wachira

**Affiliations:** 1 Food Technology Department, Auchi Polytechnic, Auchi 312001, Nigeria; 2 Nutritional Science/Dietetics Program, Morgan State University, Baltimore, MD 21251, USA; 3 Department of Biology, Morgan State University, Baltimore, MD 21251, USA

**Keywords:** millet, fermentation, microbiome, metagenomics, vitamins, biosynthesis

## Abstract

Fermented foods play an important role in the human diet and particularly so in under-resourced environments where cold preservation is not attainable due to irregular supply of electricity. Fermented foods are reported to support gut health by contributing probiotics. The purpose of this study was to investigate the microbial diversity and metabolic potential of spontaneous millet fermentation. The literature in the field was reviewed and analyses were conducted on publicly available Sequence Read Archive (SRA) datasets. Quality analysis was performed with FastQC, and operational taxonomic units (OTUs) were generated using Quantitative Insights Into Microbial Ecology (QIIME2) and Divisive Amplicon Denoising Algorithm (DADA2) pipelines with Greengenes as the reference database. Metagenomics and pathways analysis were performed with Phylogenetic Investigation of Communities by Reconstruction of Unobserved States (PICRUSt2). Statistical analysis and visualization were accomplished with Statistical Analysis of Metagenomic Profiles (STAMP). At the family taxonomic level, there were differences in the relative abundances of the dominant taxa of bacteria that are involved in the spontaneous fermentation of millet namely *Lactobacillaceae*, *Burkholderiaceae*, *Streptococcaceae*, *Leuconostocaceae*, and *Acetobacteraceae*. *Clostridiaceae* was the dominant family in one dataset. The incidence of *Lactobacillaceae* and *Bifidobacteriaceae* suggest the probiotic characteristics of fermented millet. The datasets were collected with fermentations that were mediated by autochthonous microorganisms and the presence of some potential pathogens such as *Enterobacteriaceae*, *Clostridiaceae*, *Aeromonadaceae*, *Microbacteiaceae*, *Pseudomonadaceae*, and *Neisseriaceae* which suggest the need for standardization of fermentation approaches. The genomes show the potential to synthesize metabolites such as essential amino acids and vitamins, suggesting that the respective fermented foods can be further optimized to enhance nutritional benefits.

## 1. Introduction

Food fermentation plays an important role in the human diet and it has been practiced throughout human history for the preparation and preservation of foods [1]. Fermentation naturally acidifies foods and thereby inhibits the growth of food spoilage and pathogenic bacteria [2,3]. In addition, fermentation enhances the nutritional value of food through several mechanisms including increasing the digestibility of complex carbohydrates and plant proteins, increasing the bioavailability of micronutrients, and through microbial synthesis of beneficial metabolites, such as vitamins and amino acids [4,5,6,7,8]. Microorganisms increase the protein content of food by hydrolyzing carbohydrates to synthesize proteins and, in some cases, increasing the digestible indispensable amino acid score (DIAAS), which measures the ability to utilize a protein source based on the limiting amino acids [9,10]. Microbial metabolism also increases the bioavailability of nutrients by hydrolyzing complex carbohydrates and substances that act as anti-nutrients by sequestering metabolites [11]. Grains and especially polished grains may also lack some micronutrients, and fermentation is reported to increase the levels of B vitamins and vitamin E in cereals [9,12].

Further, microbial metabolism can improve food safety by degrading harmful compounds such as mycotoxins [13]. The mycotoxins are associated with both acute toxicity, referred to as mycotoxicosis, and long-term diseases including liver cirrhosis and certain types of cancer including hepatocellular carcinoma [14,15]. They are especially detrimental to health and nutrition in Africa because the molds infect many staple grains in the fields and during storage [16]. Even in the absence of knowledge of the responsible metabolic pathways, lactic acid bacteria (LAB) that are used in fermentations are known to detoxify microtoxins through enzymatic degradation and adsorption mechanisms [15]. Thus, food fermentation could be included in strategies that are aimed at decreasing food insecurity and malnutrition.

In addition to food preservation, fermentation is reported to have health benefits arising from the microbial metabolites and an ability to augment or support the growth of beneficial gut microflora [17,18]. Thus, studies in germ-free mice indicated the beneficial role of *Bacteroides thetaiotaomicron*, a gut commensal bacterium, in stimulating normal intestinal development and function, thereby providing a potential basis for the poor growth of germ-free mice as opposed to mice that were reared in a normal environment [19]. Human studies also support the role of gut microbiome in human health and in colorectal cancer (CRC) [20]. The colorectum epithelium is largely sterile due the mucus barrier that separates it from the lumen, but microbial films colonizing the outer layer of the mucus release products that regulate the gut-associated lymphoid tissue to support a healthy epithelial barrier [20]. Through actions on the immune system, gut bacteria can enhance CRC therapy responses [20]. Conversely, some bacteria are thought to promote CRC by releasing reactive metabolites such as hydrogen sulfide and reactive oxygen species (ROS) that cause DNA damage and by promoting inflammation [20]. In addition to immunity and cancer, gut microbial metabolites are known to influence metabolism, cardiovascular function, and the nervous system [21]. At the molecular level, numerous mechanisms have been suggested to explain the properties and beneficial effects of probiotics and they generally fall under four categories [22]: the ability of the organism to survive and colonize the digestive system; the production of anti-pathogen substances, such as bacteriocins; support of the hosts immune system and promoting gut barrier functions; and the production of beneficial metabolites such as amino acids and vitamins. By identifying the genes that are responsible for these functions in probiotics and gut microflora, it may be possible to optimize the characteristics of human gut microbiota to prevent disease and promote health.

A large metagenomics study on dairy-, sugar-, and brine-based fermented foods from diverse countries demonstrated differences in microbial diversity that was largely determined by the food type and fermentation conditions [23]. Differences at the family taxonomy level was the most distinguishing feature between the food types. While differences were observed based on whether fermentation was initiated with a starter culture or spontaneously, the microbial growth substrates appear to be the main determinant of fermented food microbiomes [23]. Still, while the study identified 476 unique species in the 58 samples that were analyzed, *Lactobacillus* is the dominant genus in the fermentations. Food-specific microbiomes have also been reported with traditional foods from India [24]. The dominant taxa were *Lactobacillus* for bamboo shoots, *Staphylococcus* for soybean, and *Clostridium* for pork fat fermentations. Thus, there is a need to analyze and characterize fermentation microbiomes from different geographic regions and cultural food preparation practices.

Traditional fermentation of grains is common in many African countries; however, the lack of understanding of the microbiology that is associated with the various fermentation techniques has remained a concern [25]. Studies have identified the dominant organisms in traditional fermentation cultures as various genera of fungi, yeasts, and bacteria, including LAB and *Bacillus* [26]. *Lactobacillus, Lactococcus*, *Streptococcus*, *Enterobacter*, *Klebsiella*, and *Acetobacter* were identified in microbiomes that were associated with the non-alcoholic fermentation of grain in Zambia [27]. Similarly, in Senegal, milk that was fermented with starter cultures were analyzed and found to contain *Streptococcus* and *Lactobacillus* in all samples [28]. These two genera constituted, on average, 61.2% and 31.5%, respectively, of the total bacterial community [28]. The authors reported variability in the microbial composition of fermentation cultures based on the region of study. Similarly, microbiome studies on grain fermentation for beverages identified *Lactobacillus* as the dominant taxa of bacteria, but also reported variability in microbial diversity between different formulations [29]. Still, it is possible that less abundant organisms play significant roles in supporting the fermentations and determining the food quality.

Based on studied fermented food microbiomes, it can be concluded that different assortments of microbes facilitate the fermentation of foods, perhaps based on the overall metabolic capacity rather than the microbial composition of the cultures. The purpose of this study was to investigate potential nutrition-enhancing properties in microorganisms that are associated with traditional cereal fermentation practices.

## 2. Materials and Methods

Food insecurity remains an important problem in society that is being exacerbated by climate change; it is estimated that up to 170 million people could be at risk of hunger by 2080 [30]. Food security, as defined by the FAO and reviewed by Schmidhuber and coauthors, is a multidimensional problem that encompasses shortfalls in methods of production, distribution, storage, and access and as such it requires study at different scales [30]. This study focused on the microbiomes that are involved in the fermentation of grains that are indigenous to regions with the risk of water shortage stress. It addresses two important questions: whether the metagenomes of fermentation microorganisms have the capacity to synthesize key micronutrients and the extent of bacterial taxonomic diversity that is associated with millet fermentation. A search of the literature and the SRA database was conducted with different terms including “metagenome”, “millet”, and “fermentation” leading to the identification of three bioprojects reporting on traditional millet fermentation microbiomes as follows: PRJNA482055 [29], PRJNA532858 [31], and PRJNA662102 [32]. The relevant articles were retrieved for review. Further, additional analysis was conducted as described below.

Source of data: Datasets were obtained from National Center of Biotechnology Institute (NCBI) Sequence Read Archive (SRA) database [33]. The following datasets were selected and represent samples that were collected at the beginning of the fermentation and after 12 h: SRR7544761, SRR7544763, SRR7544764, SRR7544765, SRR7544766, SRR7544767, SRR7544768, SRR7544777, SRR7544778 [29]. Additional millet fermentation datasets were selected from the PRJNA662102 bioproject as follows: white fonio (SRR12608638, SRR12608635, and SRR12608634), brown fonio (SRR12608642, SRR12608640, and SRR12608639), and finger millet (SRR12608646, SRR12608644, and SRR12608643). The data were collected at three souring time points of 0, 48, and 72 h.

Methods: The data were analyzed using the following software packages: Qiime2 [34], DADA2 [35], PICRUSt2 [36], and STAMP [37]. Putty was used as the secure shell client to access the supercomputing clusters PSC/Bridges2 and TACC/Stampede2 [38,39,40]. Data transfers were accomplished with Globus [41]. The archived fastq reads were retrieved with the SRA toolkit [42]. The quality of the data was initially assessed with FastQC and summarized with MultiQC [43,44]. The data were then imported into qiime2 artifacts. The DADA2 pipeline was used to trim low quality regions and to construct feature tables for further analysis. The q2-phylogeny plugin was used to generate a phylogenetic tree using the align-to-mafft-fasttree pipeline [45]. Alpha and beta diversity analysis were performed with the q2-diversity plugin [46,47]. Rarefaction plotting confirmed the adequate representation of the bacterial community in the data. The q2-feature-classifier plugin that was trained with Greengenes 13_8 99% Operational Taxonomic Units (OTUs) was then used for taxonomy analysis [48,49]. Visualizations were generated with Qiime2View [34]. The studies involved 16S rRNA gene sequencing as such information on metabolic pathways is missing. PICRUSt2 utilizes a database of gene families and reference genomes to reconstruct metabolic pathways from marker genes [36]. PICRUSt2 was used to predict the metagenomes followed by biological functions that are associated with the OTUs. The OTUs were predicted with the closed-reference method using the Greengenes database v.13.5. and clustered at 97% using QIIME and vsearch [50]. The OTU tables were exported in biom format for functional analysis [51]. The OTU tables were uploaded to a Galaxy instance of PICRUSt [36,52]. They were normalized by copy number followed by the prediction of the metagenome and then clustered by function. Statistical analysis and visualization were accomplished with STAMP [22]. The unclassified OTUs were removed, and two groups, initial and final fermentation samples, were compared with a *t*-test at 95% confidence interval. OTUs with low representation were screened out during the analysis.

## 3. Results

Spontaneous fermentation is driven by environmental microorganisms and, in general terms, the variability has been attributed to substrate types [53]. However, based on cultural practices, specific microorganisms are enriched through the use of different fermentation containers and back-slopping [53]. *Lactobacillus* was identified as the most dominant genus of bacteria in millet fermentations in all three datasets (Table 1). *Enterobacteriaceae* was most abundant genus in one sample that was fermented under laboratory conditions [31]. *Acetobacter*, under *Proteobacteria*, was abundant in one sample and it comprised approximately 30% of the OTUs [29,31,32]. *Bifidobacterium* under the *Actinobacteria* phylum was also present but at lower levels in one sample as was *Chryseobacterium*, under *Bacteroidota*, which was abundant in one sample [31,32]. However, in all cases, there was a high degree of variability in the proportions of the taxa that were present in the different samples that were included in the study. In kunu fermentation, one sample was dominated by *Clostridium* in the late stage of fermentation [29]. *Enterococcus* and *Enterobacteriaceae* also occurred in varying proportions in some samples of fermented millet [31]. Fermentations that were conducted under laboratory conditions had higher proportions of *Enterococcus* and *Enterobacteriaceae*. There were two studies that reported on the reduction of mycotoxins in fermented cereals [29,32].

**Figure 1:**
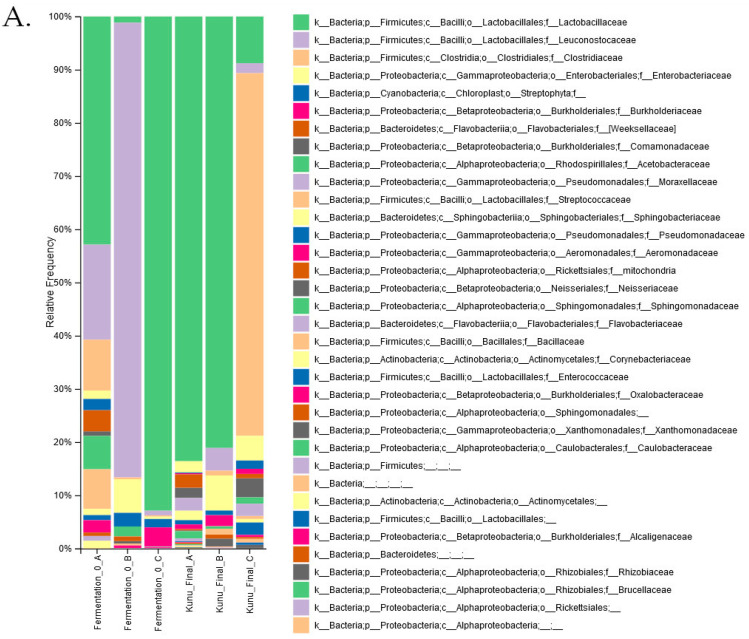

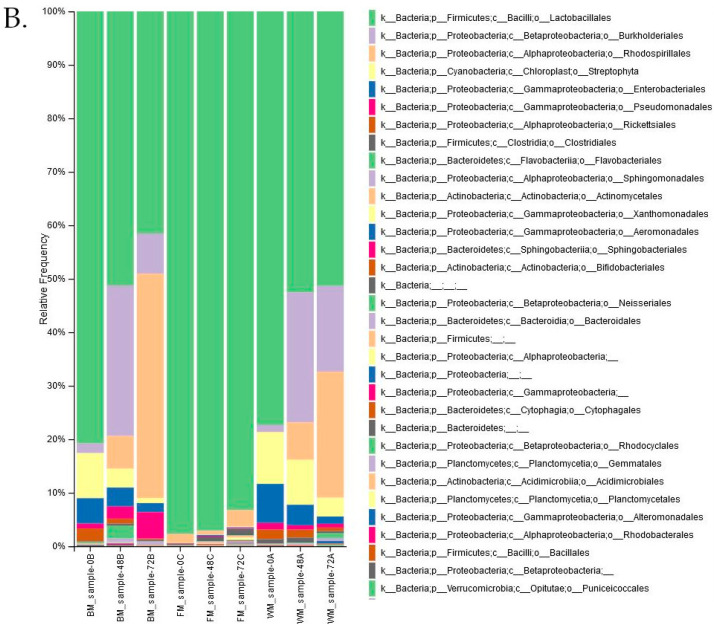
Bacterial diversity during millet beverage fermentation. Alpha diversity data were visualized at different taxonomic levels. (**A**) Kunu, three fermentations were conducted at different time points and the data shown are for 0 h and 72 h timepoints [29] and (**B**) Ogi fermentations of brown millet (BM) fonio millet (FM), and white millet (WM) [32]. The data shown are for the 0 h, 48 h, and 72 h time points. Differences are observed at the family and genus levels.

**Figure 2:**
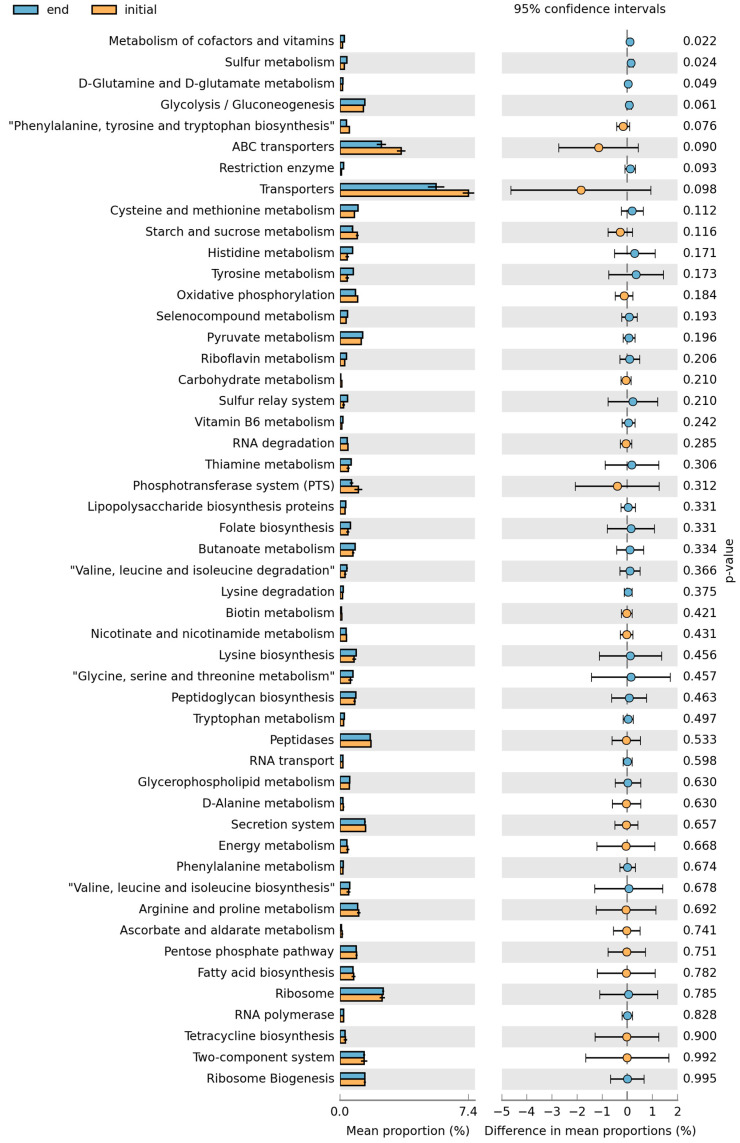
Micronutrient synthesis pathways in the kunu fermentations. The metagenomes were reconstructed and the pathways identified with PICRUSt. Pathways for the synthesis of vitamins and essential amino acids were identified in the metagenome. The figure was constructed from the fermentations A and B. Fermentation C was excluded due the high abundance of *Clostridium* that is not characteristic of cereal fermentations.

Additional analysis was based on BioProject PRJNA482055 and PRJNA662102, which are 16S rRNA gene sequencing studies of microbiomes from fermented beverages from Nigeria [29,32]. Diversity analysis was conducted with Qiime2 prior to functional analysis. *Firmicutes* were the dominant phylum in all six samples that were analyzed in the PRJNA482055 bioproject. The data were collected with spontaneous fermentations and all the samples had different representations of bacteria at the family level at time 0. At the end of the fermentation at 12 h, two samples were dominated by *Lactobacillaceae* and the third one by *Clostridiaceae* at the family taxonomy level (Figure 1A). *Lactobacillus* species dominated three samples (Figure 1A). At the functional level, pathways for the biosynthesis of various essential amino acids, including lysine and tryptophan, and branched-chain amino acids and for folate were present in the predicted metagenomes (Figure 2). The metagenomes also contain pathways for ascorbate, riboflavin, thiamin, and vitamin B_6_ metabolism. However, enrichment in the number of genes was not observed in samples from the later (12 h) time points. Bacterial diversity in other millet fermentation datasets was reanalyzed to assess potential changes in bacterial communities over the time course of beverage maturation (Figure 1B).

Cereal fermentations are accompanied by changes in the dominant species as acidification increases over time, starting with the autochthonous organisms such as *Enterobacteriaceae* that are replaced by various species of LAB and finally the dominant species are *Lactobacilli* species [53]. A reanalysis of SRA data that were collected on a time course basis during the fermentation of three millet varieties was conducted [32]. As reported, *Lactobacillaceae* constituted 92% of all OTUs in FM at the 0 h time point and this percentage decreased to 67% at 48 and 72 h. The decrease was accompanied by an increase in *Streptococcaceae* OTUs to 26% at 48 h. In BM fermentations, a decrease in *Lactobacillaceae* OTUs during fermentation from 71% at 0 h was accompanied by an increase in *Burkholderiaceae* OTUs at 48 h from 2% to 28% and an increase in *Acetobacteraceae* to 42% at 72 h. In WM, *Lactobacillaceae* OTUs accounted for 40% of all the OTUs at 0 and 48 h and decreased to 28% at 72 h. However, *Leuconostocaceae* OTUs accounted for 26% of all OTUs at 0 h and declined to 2% at 48 h with a corresponding increase in *Burkholderiaceae* OTUs to 24% from 1%. *Acetobacteraceae* increased from 0.05% at 0 h to 23% at 72 h.

## 4. Discussion

While fermentation is an ancient practice that is widely used for food preservation and to enhance quality, the relevant food microbiomes are increasingly attracting attention to better optimize the techniques that are used and due to the known health benefits of fermented foods [54]. In addition to inhibiting the growth of pathogenic microbes, fermentation is reported to degrade mycotoxins [29,32,54]. In addition to reviewing the literature on millet fermentation, this report reanalyzed SRA data using the DADA2 pipeline and the Greengenes 16S rRNA database, which generated suitable data for PICRUSt analysis. In agreement with the original study [29], variability in taxa that are associated with different fermentation batches was noted. Known food fermentation bacteria such as *Lactobacillus*, *Leuconostoc*, *Pediococus*, and *Streptococcus* were among the most abundant genera in two of the three fermentations that were examined (Figure 1). The presence of *Enterobacteraceae* in the bacterial community of one fermented product could be an indication of contamination and further suggests the need to develop suitable and well characterized starter cultures and processes for the fermentation of traditional beverages. The feasibility of such an approach has been demonstrated with cowpea leaf fermentation in which parameters were optimized to achieve desired end-points of titratable acidity and microbial composition [55]. Still, genomics studies have a limitation in that the presence of viable organisms is not quantified and as the reported OTUs or amplicon sequence variants, ASVs, may not necessarily indicate the existing microorganism at the time of sampling [53].

Analysis of the predicted metagenomes did not reveal significant differences in the abundance of pathways for nutrient synthesis between the initial and final fermentations (Figure 2). This is despite obvious differences in the abundances of bacteria genera between the initial and final stages of fermentation (Figure 1). Thus, metabolite measurements using throughout metabolomics are needed to further investigate the potential accumulation of desired metabolites in fermented foods and the reduction of potentially contaminating mycotoxins. However, many micronutrients and essential amino acid metabolism pathways were identified with PICRUSt2 (Figure 2). This is consistent with the reported nutritional benefits of fermented cereals [23,24]. Fermentation of cereals by LAB increases the nutritional value through the production of B vitamins (including folate), vitamin K, and amino acids such as lysine. Fermentation also enhances iron utilization through the breakdown of complex substances that sequester micronutrients and reduce bioavailability [2].

The role of the gut microbiome in health and disease has been reported in many studies [56]. Gut microorganisms produce many beneficial metabolites in a mutualistic relationship with the host and changes are associated with many diseases including inflammatory bowel disease, cancer, and neurological and cardiovascular diseases [56,57]. While questions remain as to how the diet influences the gut microbiota, some microorganisms that are associated with fermented food are termed as probiotics and are primarily LAB [58]. The probiotic term implies that the organisms promote a healthy gut microbiota without necessarily becoming resident in the host. The bacteria that were identified in this dataset, including LAB, are known probiotics.

The study focused on grains that are used as food sources in different parts of Africa. Many regions of the world, and especially Africa, face the risk of food insecurity as a result of climate change [59,60]. This necessitates research into plant biology with a goal of understanding the scientific basis for resiliency to biotic and abiotic stresses [59].

The native grasses that supply grains in many countries in Africa are an important genetic resource that could help reduce factors that are associated with food insecurity [61]. Notably, the majority of countries with a Global Hunger Index of serious to alarming are in Africa and Asia [60]. In a survey dubbed as the lost foods of Africa, over 100 native grasses, the lost grains of Africa, were identified among over 2000 edible plants that are well adapted to the African climate and have served as food sources for humans over thousands of years [61]. Among these, the currently cultivated grains include finger millet (*Eleusine coracana*) and fonio (*Digitaria exilis* and *Digitaria iburua*). The fermentation of these grains increases quality by reducing harmful mycotoxins and potentially enhancing nutritional value.

An interesting observation has been that the composition of food fermentation microbiomes is largely determined by the type of food [23]. Thus, dairy microbiomes are dominated by *Lactococcus lactis* and *Streptococcus thermophilus* with 44.8% and 16% of the reads, respectively; brine-based fermentations had *Lactobacillus* being represented by 48% of the reads; and *Proteobacteria* accounted for 44.8% of reads in sugar-foods. The data on millet fermentation suggest the abundance of *Lactobacillus* and to a lesser extent *Pediococcus* and *Enterococcus* (Table 1). The reasons for the differences in microbial compositions that were observed in the fermentation mixtures of the three varieties of millet remain unknown. Still, the mechanistic reasons for these differences require further exploration as a basis for guiding the design of synthetic microbial communities with desired food improvement characteristics.

While the reviewed studies focused on bacteria taxa, molds and yeasts are also important in food fermentation and further studies are needed to understand their potential role in millet fermentation microbiomes. The role of fermentation is likely to continue to play a significant role in human nutrition as it enhances nutritional, taste, and texture qualities of diverse foods [9]. Still, some microorganisms lead to food spoilage that is characterized by bad odors, toxins, and food-related illnesses [9]. Improperly stored grains support the growth of mycotoxin-producing fungi that are harmful to human health [9]. Importantly, millet fermentation is reported to reduce the levels of mycotoxins in fermented cereals [29,32]. Still, many unanswered questions remain, including on the source of the fermenting microorganisms and how that is affected by the quality of the substrates and water utilized; the basis of the sequelae of microorganism colonization and growth in millet preparations; and the constituent metabolites in the end products and how they might influence human health.

## 5. Conclusions

This study reviewed the literature on millet fermentation and reanalyzed existing datasets to determine the potential to produce micronutrients. In sum, *Lactobacillaceae*, *Burkholderiaceae*, *Streptococcaceae*, and *Acetobacteraceae* are the predominant bacteria in millet fermentations and the predicted metagenomes include vitamin biosynthesis pathways. While no enrichment in these pathways is observed in the final stages of fermentation, food fermentation is known to enhance food nutritional value. Given that 16S ribosomal RNA amplicon sequencing as well as metagenomics datasets have been collected on fermented foods from different countries and encompassing the main regions of Africa [13], additional analyses followed by experimental investigations could lead to the engineering of microbial communities that confer desired characteristics to fermented foods.

## Figures and Tables

**Table 1:** Genera of LAB that were detected in millet fermentation microbiomes. The asterisks indicate the most abundant genera with each asterisk representing one sample. A plus symbol indicates the presence of a genus in at least one sample.

Lactobacillales Genus	Diaz et. al. [31]	Ezekiel et. al. [29]	Chibuzor-Onyema et. al. [32]
*Lactobacillus*	*****	**	********
*Lactococcus*	+	+	+
*Leuconostoc*	+		+
*Pediococcus*	+	+	****
*Streptococcus*	+		+
*Aerococcus*			
*Alloiococcus*			
*Carnobacterium*			
*Dolosigranulum*			
*Enterococcus*	**		
*Oenococcus*			
*Tetragenococcus*			
*Vagococcus*			
*Weissella*	+	+	*

## Data Availability

The data analyzed in this study were obtained from the SRA database and are archived under the following accession numbers: PRJNA482055, PRJNA532858, and PRJNA662102.
